# Rasl11b Knock Down in Zebrafish Suppresses *One-Eyed-Pinhead* Mutant Phenotype

**DOI:** 10.1371/journal.pone.0001434

**Published:** 2008-01-16

**Authors:** Guillaume Pézeron, Guillaume Lambert, Thomas Dickmeis, Uwe Strähle, Frédéric M. Rosa, Philippe Mourrain

**Affiliations:** 1 INSERM, U784, Paris, France; 2 Ecole Normale Supérieure, Paris, France; 3 Max Planck Institute for Developmental Biology, Tübingen, Germany; 4 Institute for Toxicology and Genetics, Forschungszentrum Karlsruhe, Karlsruhe, Germany; Baylor College of Medicine, United States of America

## Abstract

The EGF-CFC factor Oep/Cripto1/Frl1 has been implicated in embryogenesis and several human cancers. During vertebrate development, Oep/Cripto1/Frl1 has been shown to act as an essential coreceptor in the TGFβ/Nodal pathway, which is crucial for germ layer formation. Although studies in cell cultures suggest that Oep/Cripto1/Frl1 is also implicated in other pathways, *in vivo* it is solely regarded as a Nodal coreceptor. We have found that Rasl11b, a small GTPase belonging to a Ras subfamily of putative tumor suppressor genes, modulates Oep function in zebrafish independently of the Nodal pathway. *rasl11b* down regulation partially rescues endodermal and prechordal plate defects of zygotic *oep^−/−^* mutants (Z*oep*). Rasl11b inhibitory action was only observed in *oep*-deficient backgrounds, suggesting that normal *oep* expression prevents Rasl11b function. Surprisingly, *rasl11b* down regulation does not rescue mesendodermal defects in other Nodal pathway mutants, nor does it influence the phosphorylation state of the downstream effector Smad2. Thus, Rasl11b modifies the effect of Oep on mesendoderm development independently of the main known Oep output: the Nodal signaling pathway. This data suggests a new branch of Oep signaling that has implications for germ layer development, as well as for studies of Oep/Frl1/Cripto1 dysfunction, such as that found in tumors.

## Introduction

Endoderm and mesoderm germ layer formation in vertebrate embryos requires the activity of the conserved TGFβ/Nodal signaling pathway [1], [2], [3], [4], [5], [6]. Extensive studies in mouse, Xenopus and zebrafish have allowed the establishment of the current TGFβ/Nodal pathway: Nodal signaling is activated by the interaction of Nodal ligands with activin (Alk4/ActR-IIB/Taram-A) receptors [7], [8], [9], [10], [11], [12] and is regulated extracellularly by an EGF-CFC coreceptor (see below) and by antagonists belonging to the TGFβ/Lefty family [13], [14], [15], [16]. Nodal signaling is then transmitted intracellularly by phosphorylation of Smad2, and Smad2-P translocation in the nucleus in turn induces the expression of conserved mesendodermal transcription factors (see [17], [18] for review).

Mesendoderm induction by Nodal signaling requires a conserved EGF-CFC factor encoded by the gene *Cripto1* in mouse [19], [20], *Frl1 (Fgf receptor ligand 1)* in Xenopus [21] and *one-eyed-pinhead* (*oep*) in zebrafish [22], [23]. The function of Oep/Cripto1/Frl1 as a coreceptor of the Nodal pathway has been clearly demonstrated. In mouse, *cripto* mutants fail to form embryonic mesendoderm and resemble the mouse *nodal* mutant [10], [19], [24], [25]. Similarly, in zebrafish, embryos lacking both maternal and zygotic *oep* function (MZ*oep*, obtained by crossing rescued *oep−/−* adult mutants [22]) display a phenotype similar to the zebrafish *cyclops;squint* (*cyc;sqt*) nodal double mutant [22]. They have an alterated antero-posterior axis and fail to develop endoderm, posterior mesoderm and prechordal plate (PP, a mesendodermal structure). More recently, biochemical analyses have shown that Oep/Cripto1/Frl1 is required for Nodal ligand binding to the Alk4/ActR-IIB/Taram-A receptor complex and subsequent Smad2 phosphorylation [10], [26], [27].

Other signaling pathways may also require the participation of EGF-CFC molecule either as a cofactor or as a ligand. For instance, in Xenopus and zebrafish, Frl1/Oep could function as antagonist of BMP [28] and more recently, in Xenopus, Frl1 has been implicated in the Wnt pathway and also been shown to be a coreceptor for Wnt11 [29]. Moreover, in cell culture, a soluble form of Cripto1 has been shown to bind the receptor Glypican1 and activate both the ras/raf/ERK/MAPK and PI3-K/AKT/GSK-3β intracellular signaling pathways, which are both well known for their involvement in oncogenic processes [30], [31], [32], [33]. Interestingly, Cripto1 has been found in the conditioned medium of several human carcinoma cell lines suggesting that a naturally soluble isoform of Cripto1 could act as a diffusible ligand [34], [35], [36]. Thus, several reports suggest a multifunctional capability, both as ligand and as coreceptor of Oep/Cripto1/Frl1. The role of Oep/Cripto1/Frl1 in pathways other than Nodal *in vivo* is still a matter of debate, certainly due in part to this complexity. The identification of additional elements which respond to Oep signaling stands to elucidate important signaling outputs of this protein.

Here we provide genetic data which supports that *oep* can influence mesendoderm formation independently of Nodal signaling. We report the analysis of Rasl11b and the first developmental function for a member of the Rerg/Rasl11-12 subfamily [37], [38], [39], [40], [41]. We find that *rasl11b* down regulation acts as a specific suppressor of the *oep* phenotype and partially rescues endoderm and prechordal plate formation in *oep* deficient embryos. Further, Rasl11b activation inhibits mesendoderm formation only when *oep* is down regulated, and not in wild-type embryos. Interestingly, *rasl11b* loss of function does not rescue mesendoderm formation in other mutants within the Nodal pathway and does not affect the activation level of Nodal signaling as assayed by phosphorylated Smad2 monitoring. This genetic interaction between *oep* and *rasl11b* strongly suggests that Oep can have a Nodal-Smad2 independent influence on mesendoderm formation. This study has implication for endoderm and mesoderm germ layer development but also potentially for analysis of Oep/Frl1/Cripto1 dysfunction, such as that found in tumors.

## Results

### Rasl11b is a cytosolic small GTPase

**Figure 1:**
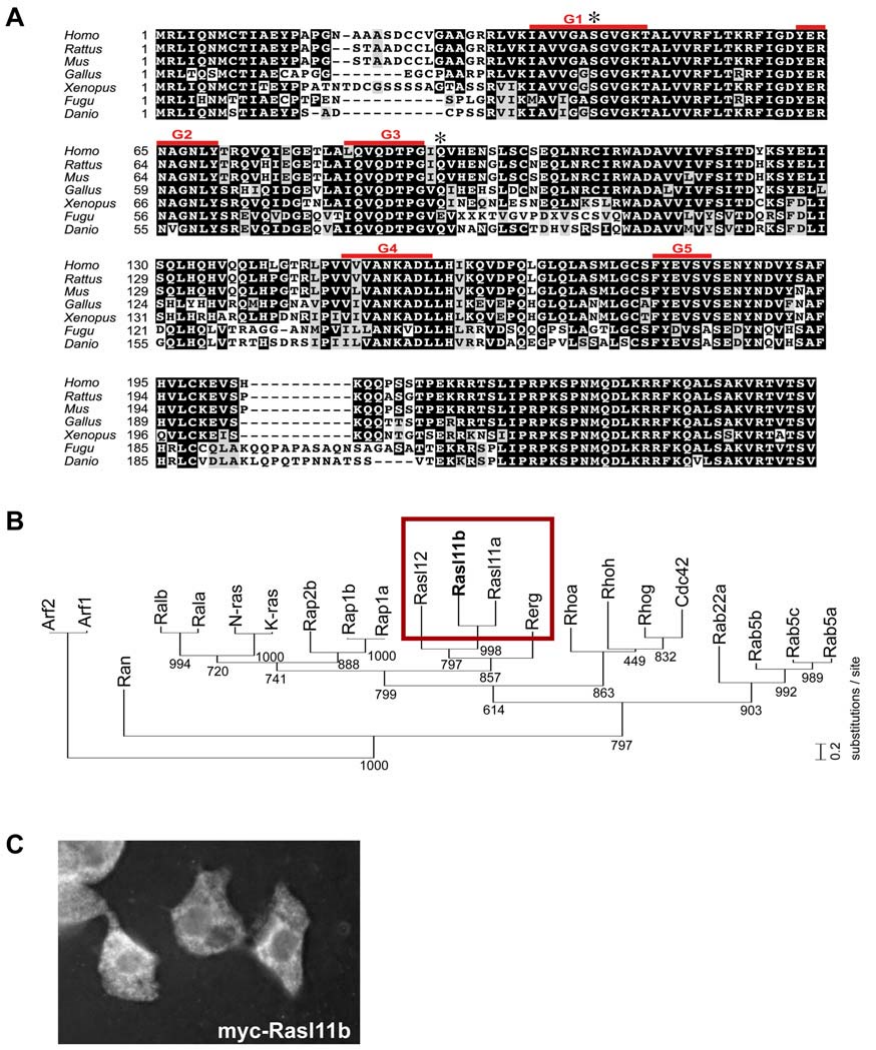
Rasl11b, an atypical cytoplasmic Ras small GTPase, is strongly conserved in Vertebrates. (A) Zebrafish *rasl11b* encodes a Ras-related small GTPase of 244 amino acids (accession number DQ983377) containing the 5 highly conserved domains (G1–G5, overlined in red) responsible for the guanine nucleotide-dependent molecular switches. Rasl11b has no obvious orthologues in *Drosophila melanogaster* or *Caenorhabditis elegans*, but is highly conserved among vertebrates. Note that, in contrast to most of the Ras small GTPases, Rasl11b lacks a COOH-terminal CAAX motif and any known recognition signal for C-terminal lipidation found in Ras proteins such as farnesylation or palmitoylation allowing membrane anchorage. The amino acid positions mutated to create the activated forms Rasl11b^S42V^ and Rasl11b^Q82L^ are indicated with stars. (B) Phylogenic analysis of zebrafish small GTPase proteins. The degree of relatedness is indicated by the length of the vertical lines. Numbers indicate bootstrap support for nodes. Red box: Rasl11b, Rasl11a, Rasl12 and Rerg constitute an uncharacterized branch of Ras proteins devoid of lipid modification signals. (C) Epifluorescent microscopy of zebrafish embryonic cells expressing myc-Rasl11b revealed by immunostaining.

*rasl11b* cDNA has been isolated from a subtractive library between embryos expressing a constitutively activated Nodal signaling pathway and control embryos [42]. Activation of the Nodal pathway forces most blastomeres to adopt a mesendodermal fate [43], [44], so this strategy was designed to isolate either Nodal pathway downstream components or other genes specifically expressed in mesendodermal territories. This library previously allowed us to isolate the endodermal gene *casanova* [42] and to integrate it in the Nodal transcriptional cascade [45]. The Rasl11b protein is highly conserved among vertebrates (Fig. 1A), sharing on average 94% homology with its mammalian orthologues. Rasl11b, along with Rerg [39], Rasl11a [38] and Rasl12/Ris [41], constitute a poorly documented group of the Ras small GTPase family [40] (Fig. 1B). Like other members of this particular branch of GTPases, Rasl11b lacks any known carboxy-terminal recognition site (such as a CAAX box, A = aliphatic, X = terminal amino acid) for post-translational lipid modification [39], [40], [46], which is used in most Ras small GTPases to allow anchorage to the plasma membrane (Materials and Methods, and Fig. 1A). Consistent with this, embryos expressing myc-Rasl11b revealed no accumulation at the plasma membrane but rather a distribution in the cell cytoplasm (Fig. 1C).

### Rasl11b has a maternal and a zygotic component

**Figure 2:**
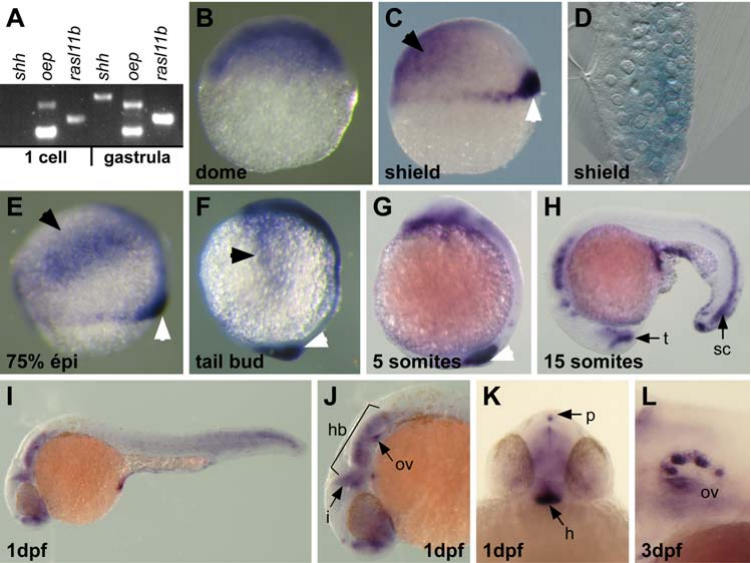
* rasl11b* expression pattern during zebrafish embryogenesis. (A) RT-PCR analysis showing that *rasl11b* has a maternal and a zygotic component. RNA extractions have been done before (1 cell stage) and after (gastrula) the midblastula transition, the time of activation of the zygotic transcription. The maternally and zygotically expressed *oep* gene and the strictly zygotic *sonic hedgehog* (*shh*) gene have been used as controls. (B) During the cleavage period (dome stage), *rasl11b* is ubiquitously expressed. (C) At the onset of gastrulation (shield stage), *rasl11b* is still detected at the animal pole (Black arrowhead) but is also expressed in a dorso-ventral gradient at the dorsal margin (white arrowhead). This marginal expression overlaps with the mesendodermal territory in zebrafish embryos. (D) Sagittal section. *rasl11b* transcript accumulates in both hypoblastic and epiblastic dorsal blastomeres. (E, F) *rasl11b* expression is maintained at the margin throughout gastrulation (white arrowheads). Gastrulae also expressed *rasl11b* mRNA in ectodermal precursors located at the lateral borders of the blastoderm (black arrowheads). (G–K) During somitogenesis and organogenesis, *rasl11b* is expressed in the tail tip and the spinal cord (sc), and in several head structures such as the hindbrain (hb), the isthmic organizer (i), the otic vesicle (ov), the pineal gland (p), the ventral hypothalamus (h) and the posterior boundary of the telencephalon (t). (J) lateral close up and (K) frontal view. (L) At 3 dpf, *rasl11b* is no longer expressed except in the otic vesicle (lateral close up).

In zebrafish embryos, we found that *rasl11b* was first maternally expressed and transcripts were homogenously distributed in the blastoderm during the segmentation stage, as detected using RT–PCR and ISH (Figs. 2A–B). Then, throughout gastrulation *rasl11b* displayed a more restricted zygotic expression at the dorsal margin (Figs. 2C–F, white arrowheads) and in a bilateral domain at the animal pole corresponding to the border of the neural plate (Figs. 2C–F, black arrowheads). During the entire gastrulation period, *rasl11b* mRNA was detected in a dorso-ventral gradient at the dorsal margin and sagittal section revealed expression in both the hypoblast and epiblast layers at the margin (Fig. 2D). The *rasl11b* expression pattern at the margin coincided with presumptive mesendodermal territories including the dorsal organizer [47], [48]. Later, during somitogenesis and organogenesis, *rasl11b* mRNA was detected in the tailbud and tailtip, the posterior spinal chord and several head structures including forebrain, isthmic organizer, hindbrain, hypophysis and pineal gland (Figs. 2G–K). Finally, at 3 day post-fertilization (dpf) *rasl11b* expression was only observed in the otic vesicle (Fig. 2L). Thus, this dynamic expression pattern could allow *rasl11b* to function in very early development events as well as in late organogenesis processes. Most importantly, for our studies, the dorso-marginal expression suggests a putative function in endoderm and/or mesoderm germ layer formation.

### rasl11b is expressed in mesendodermal cells and controlled by the Nodal pathway

**Figure 3:**
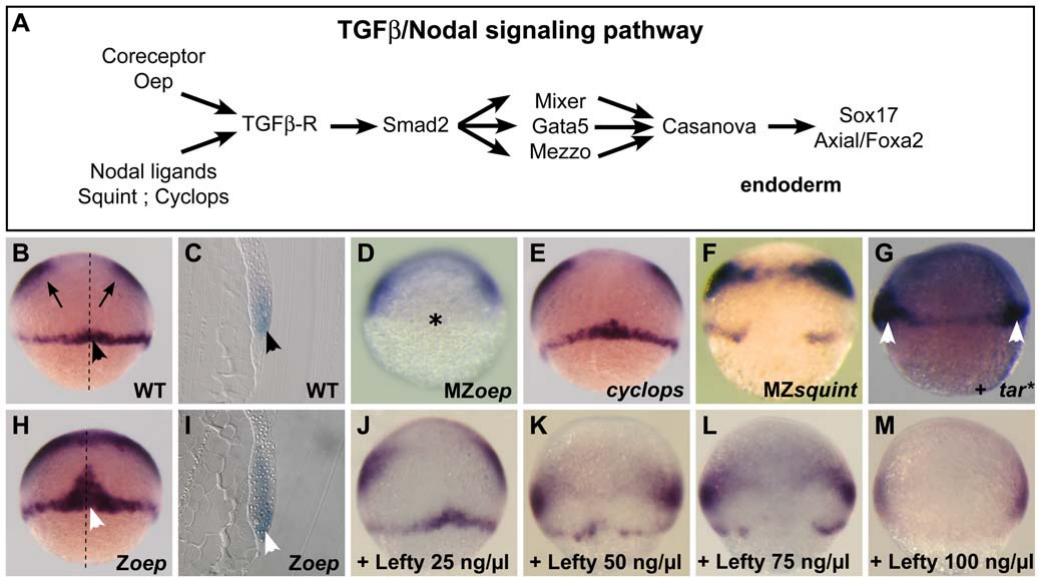
Opposite effects of Nodal and Oep on *rasl11b* expression. (A) Scheme of the Nodal cascade. The Oep coreceptor is necessary for the binding of the Nodal ligand Cyc and Sqt to the TGFβ receptors (containing the type I receptor Taram-A, the zebrafish Alk4 orthologue) that in turn phosphorylate Smad2. Smad2-P is translocated to the nucleus and triggers the transcription of a first set of genes encoding the transcription factors, Mixer, Gata5 and Mezzo. This set is required for the expression of the Sox factor Casanova that in turn initiates the transcription of the endodermal markers *sox17* and *axial*/*foxa2*. (B–M, Dorsal view of gastrulae save C, I, sagittal sections and G, lateral view) (B, C) In wild-type (WT) embryos *rasl11b* is expressed at the animal pole (arrows) and at the dorsal margin (black arrowheads). (D) This marginal expression is lost in MZ*oep* mutants (star), devoid of maternal and zygotic *oep*, consequently devoid of Nodal signaling, and so, unable to form most of the mesendoderm. (E, F) *rasl11b* dorso-marginal expression is normal in the *cyclops* Nodal mutant and largely reduced in the MZ*squint* Nodal mutant. (G) Activation of the Nodal signal by injection of a constitutively activated form of the Nodal type I receptor Taram-A (tar*) leads to a duplication of the *rasl11b* marginal expression domain (likely by inducing a second organizer, white arrowheads) (H, I) In Z*oep* mutants, devoid of zygotic *oep*, this mesendodermal expression domain is extended (white arrowheads). (J–M) A large series of embryos expressing different levels of Nodal signal was generated by injecting between blastula cells increasing doses of the recombinant Lefty protein, a Nodal pathway extracellular inhibitor. Here, only four representative doses are displayed. A progressive decrease of *rasl11b* marginal expression but no expansion was observed, even with concentrations of Lefty able to mimic a Z*oep*-like phenotype.

We then tested whether *rasl11b* putative mesendodermal expression could be modulated by Nodal pathway members (see pathway Fig. 3A). As expected, the *rasl11b* marginal expression domain (Figs. 3B,C) was completely lost in the Nodal signaling-deficient MZ*oep* mutant (lacks both maternal and zygotic components of *oep*, Fig. 3D), which fails to form mesendoderm [22]. We further examined this dorsal expression domain in the nodal mutants *cyc* and MZ*sqt* (lacks both maternal and zygotic components of *sqt*) that have intermediate Nodal signaling levels [5], [6], [49], [50]. *rasl11b* marginal expression was unaffected in *cyc* gastrulae (Fig. 3E) but was reduced in MZ*sqt* gastrulae (Fig. 3F). Besides, no change in *rasl11b* expression was observed in the Nodal downstream transcriptional effector mutants or morphants, *bonnie and clyde/mixer* (*bon*) [51], *faust/gata5* [52], *mezzo* [53], *casanova/sox32* (*cas*) [42] and *monorail/foxa2* [54] (data not shown). Finally, we tested whether activation of Nodal signaling could induce *rasl11b* expression *in vivo*. As expected, wild-type embryos injected with mRNA encoding the activated form of the Nodal receptor Tarama (Tar* [43]) displayed ectopic expression of *rasl11b* (Fig. 3G). Altogether these results show that *rasl11b* is expressed in mesendodermal dorsal territories and its expression requires the Nodal signaling pathway.

### oep has the opposite influence from nodal on rasl11b dorsal expression

During epistatic analyses, we noticed that zygotic *oep* (Z*oep*) mutant embryos (*oep−/−* embryos only containing maternally-expressed Oep) displayed a dramatic increase of *rasl11b* expression in the dorsal domain (Fig. 3H, white arrowheads). This increase corresponds to an expansion in the mesodermal layer in the antero-posterior (A-P) axis (compare sagittal sections, Figs. 3C,I). This result was surprising since Z*oep* phenotype resembles to *cyc* and MZ*sqt* phenotype's and is supposed to be due to a reduction of Nodal signaling. This suggested either *rasl11b* expression in mesoderm could be restricted by Oep in a manner different from Nodals, or Z*oep* embryos could have a particular Nodal signal level, different than in Nodal mutants, that had the opposite impact on *rasl11b* expression. To discriminate between these two possibilities and precisely investigate how Nodal signaling might affect *rasl11b* expression, we generated a large panel of embryos expressing different levels of Nodal signal by injecting different concentrations of the Nodal extracellular inhibitor Lefty [13], [14], [15], [16]. Interestingly, we observed that the inhibition of Nodal signaling with increasing doses of Lefty leads to a progressive reduction of *rasl11b* marginal expression (Figs. 3J–M, here only four representative doses are displayed). Even with Lefty doses able to mimic the *oep* phenotype at 24 hour post-fertilization (hpf), we never observed any overexpression of *rasl11b.* This data indicates that the expansion of *rasl11b* dorsal expression is a specificity of the Z*oep* mutant and is not due to a reduction of Nodal signaling but to the loss of function of *oep* and suggests that *oep* may influence mesendoderm formation independently of Nodal signaling.

### Rasl11b zygotic component has a subtle influence on antero-posterior axis formation

**Figure 4:**
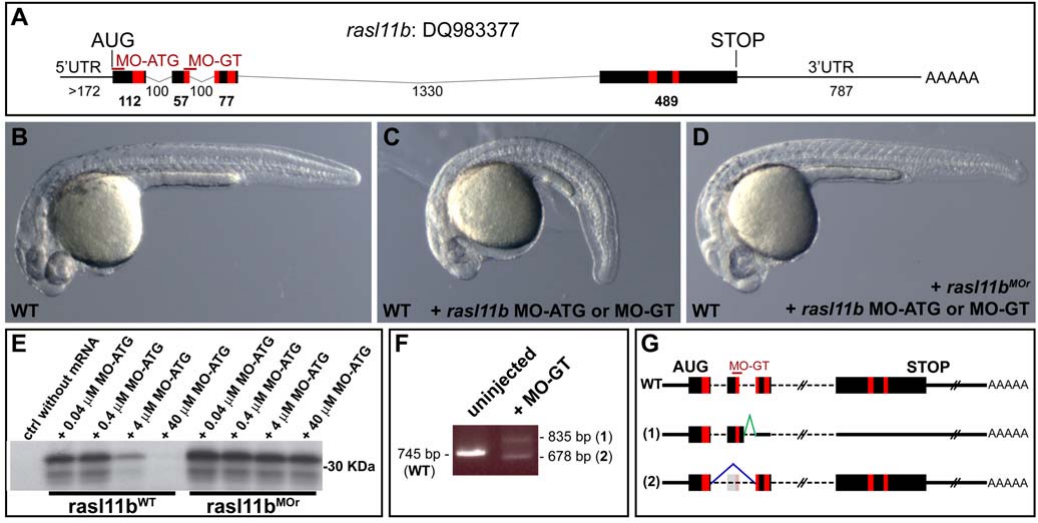
* rasl11b* knock down induces a specific curly down phenotype in zebrafish. (A) Scheme of the *rasl11b* transcript. The Morpholino targeted sequences are represented by thin red lines. The red boxes correspond to the G1 to G5 domains described in Fig. 1. (B–D) Lateral view of 1 dpf embryos. Both *rasl11b* MO-ATG and *rasl11b* MO-GT induce a curly tail down phenotype when injected in WT. This phenotype can be rescued by the coinjection of *rasl11b* mRNA resistant to MO knock down, *rasl11b^MOr^* (D). (E) In vitro translational assay showing the efficient knock down effect of the *rasl11b* MO-ATG on wild-type *rasl11b* mRNA compared to the MO resistant *rasl11b^MOr^* mRNA. (F,G) *rasl11b* splice-blocking morpholino (*rasl11b* MO-GT) knocks down endogenous *rasl11b* mRNA. RT-PCR analysis detected two splice variants that were cloned and sequenced. Sequence comparison revealed that the splice variant (1) resulted from aberrant splicing to an upstream cryptic slice donor site that is present in intron 2, resulting in an early frame shift. The splice variant (2) only missed exon 2 but is not functional (data not shown).

Rasl11b function was next investigated using morpholino (MO) knock down and mRNA misexpression experiments. MOs directed against a gene's first codon (MO-ATG) blocks translation of both maternal and zygotic expression, while MOs directed against a splice donor site (MO-GT) blocks pre-mRNA maturation, thus blocking only zygotic expression (*rasl11b* maternal mRNAs are not targeted, Fig. 4A) [55]. Wild-type embryos injected with either type of *rasl11b* morpholino displayed a curly down phenotype by 24 hpf but no missing structures (Figs. 4B,C). This phenotype was specific, as it was rescued by injecting *rasl11b* mRNA resistant to MO knock down (Materials and Methods, Fig. 4D), while *rasl11b* MO potency was confirmed using translational and RT-PCR assays and the two spliced variants produced after MO-GT injection were shown to be null as they both were unable to rescue this curly down phenotype (Figs. 4E–G and data not shown). Zygotic *rasl11b* is continuously expressed in the dorsal margin, the tail bud and tail tip throughout gastrulation and somitogenesis (Figs. 2C–H, white arrowheads), and therefore, could actually be involved in the proper organization of the trunk and tail. However, the absence of loss of structures in the *rasl11b* morphant embryos rather suggests a subtle role in A-P axis formation.

### rasl11b knock down partially suppresses oep phenotype

**Figure 5:**
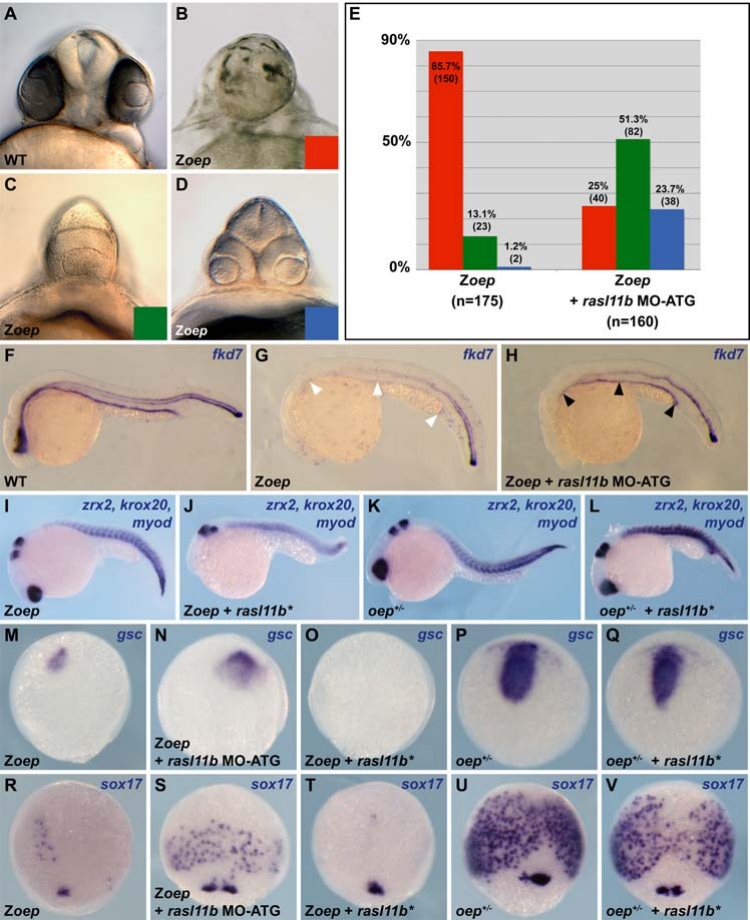
Rasl11b inhibits endoderm and PP formation in an Oep deficient background. (A–D) Frontal views of 1 dpf wild-type embryo and Z*oep* embryos. Depending on the quantity of *oep* mRNA accumulated during oogenesis, Z*oep* embryos display different levels of anterior structure development: from absence of lens and forebrain (red class, B), via a cyclopia phenotype (green class, C) to two separate retinae (blue class, D, very rare). In each clutch, these categories can have slightly different proportions depending on the female, but a large majority of Z*oep* embryos do not even develop one retina. Whatever females (n>10) were used for the *rasl11b* knock down experiments, a drastic rescue (>70%) of the forebrain formation was always observed, and a large category of embryos with two retinae appeared. (E) Cumulated numbers for each class from 5 independent *rasl11b* MO-ATG knock down experiments. (F–H) *forkhead7* (*fkd7*) expression pattern in 1 dpf embryos. Gut defects (white arrowheads) are also rescued (black arrowheads) in Z*oep* embryos injected by *rasl11b* MO-ATG. (I–L) 1 dpf embryos lateral views. Rasl11b constitutively activated forms (Rasl11b*) prevent head formation and disturb the antero-posterior axis formation in Z*oep* and *oep* ^+/−^ but not in wild-type embryos (not shown). *zrx2* is expressed in the retina, *krox20* in rhombomeres 3 and 5, *myod* is expressed in the somitic mesoderm. (M–V) dorsal view of late gastrulae. *rasl11b* knock down rescues expression of the prechordal plate marker *goosecoid* (*gsc*) and the endodermal marker *sox17* in Z*oep* whereas Rasl11b* reduces their expression in Z*oep* and *oep* ^+/−^ embryos.

*rasl11b* overexpression in the Z*oep* mutant revealed a particular genetic interaction between *oep* and *rasl11b*. We next analyzed this interaction by blocking Rasl11b function in Z*oep* mutant (As for the epistatic analysis described above, we used the *oep^tz57^* null allele [23]). Z*oep* mutants display strong gut and forebrain defects at 24 hpf [56], [57]. However, as mentioned above *oep* is maternally expressed, thus depending on the oocyte's accumulation of maternal *oep* mRNA and protein, Z*oep* larvae have variable head phenotypes. These phenotypes can be classified in three categories: 1) absence of forebrain and lens structures, 2) only one lens and 3) two fused lenses (Fig 5B–D). Strikingly, eliminating maternal and zygotic components of *rasl11b* using MO-ATG partially rescued anterior defects found in Z*oep* mutants. Indeed, whereas most of the control embryos did not develop any lens nor forebrain (85.7%), most of Z*oep*;*rasl11b* MO-ATG larvae displayed a better forebrain development and had one (51.3%; Fig. 5E) or two lens (23.7%; Fig. 5E). In addition to the head structure improvement, the gut phenotype in Z*oep* mutants was also partially rescued by knock down of *rasl11b* (Figs. 5F–H). This suppression of phenotype was also observed in *oep* morphants injected with *rasl11b* MO-ATG, dismissing any allele-specific interaction between *rasl11b* and *oep* (data not shown).

**Figure 6:**
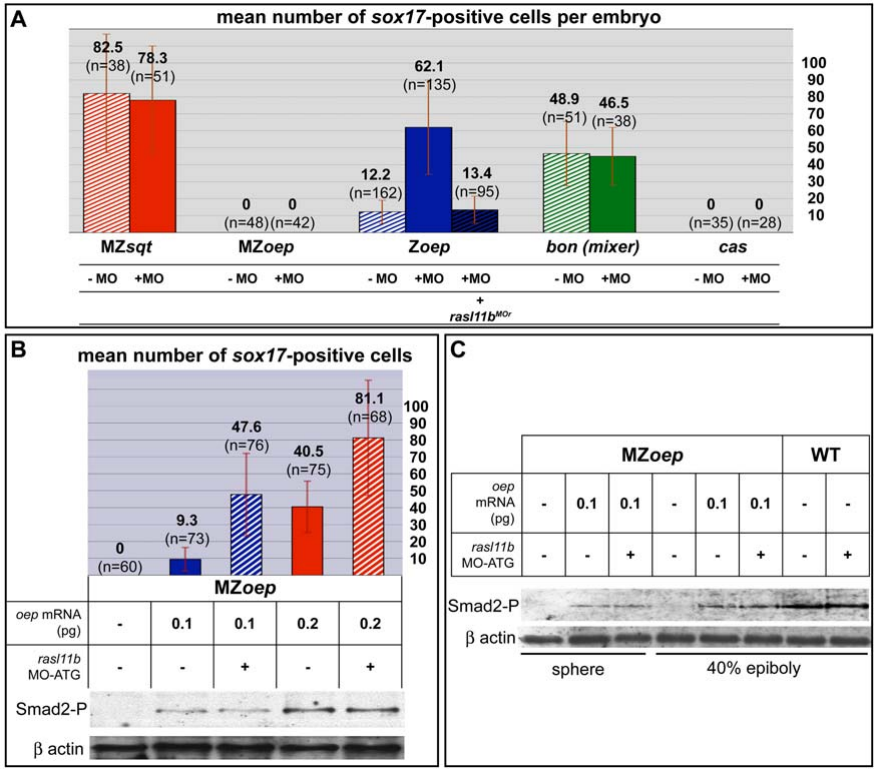
* rasl11b* interacts specifically with *oep* but does not affect the Nodal/Smad2 transduction pathway. (A) The MZ*sqt*, MZ*oep*, Z*oep*, *bon* and *cas* mutants have a clear reduction of endodermal cells and so were used to quantify the putative impact of *rasl11b* knock down at different steps/levels of the nodal pathway. The Z*oep* mutant was the only one rescued by the *rasl11b* MO-ATG injection. This rescue was abolished by co-injection of *rasl11b* MO-resistant mRNA (*rasl11b^MOr^*). Error bars indicate standard deviation. (B, C) It is impossible to generate clutches of 100% Z*oep* embryos, and because one embryo cannot give enough material for both immunoblot and genotyping experiments, 100% Z*oep-*like mutant clutches were produced by injecting clutches of 100% MZ*oep* eggs with low doses of wild-type *oep* mRNA. Half of them were then co-injected with *rasl11b* MO-ATG. Each batch was split in two, one used for phosphorylated Smad2 (Smad2-P) level analysis, the second for endodermal cell number count (assayed by sox17 in situ hybridization). Proteins were detected by western blotting using a Smad2-P antibody. Note that increasing doses of wild-type *oep* mRNA were correlated with an increase of Smad2-P and *sox17* endodermal cell number, whereas co-injection with *rasl11b* MO-ATG increased endodermal cell number without generating more Smad2-P (Error bars indicate standard deviation). The same experiment was done at three pregastrula stages: dome (B), sphere and 40% epiboly (C).

The Z*oep* phenotype observed at 24 hpf has been shown to be due to a dramatic reduction of the prechordal plate (PP) and endoderm formation during gastrulation [56], [57]. We thus analyzed PP formation and endodermal cell number in *rasl11b* MO-ATG injected Z*oep* gastrulae. In all these gastrulae, the PP was extended (Figs. 5M,N), while the endodermal cell number was up to 10 times as high as in the controls (Figs. 5R,S). Moreover, this rescue was inhibited by the co-injection of the MO-resistant mRNA of *rasl11b* confirming the specificity of our MO (Fig. 6A and data not shown). Thus, *rasl11b* MO-ATG mediated knock down suppresses a large fraction of Z*oep* early defects and hence demonstrates that Rasl11b function is partially responsible for Z*oep* phenotype. Interestingly, in contrast to the rescue observed with the *rasl11b* MO-ATG, we never observed any rescue in *rasl11b* MO-GT injected Z*oep* embryos. These results point out an important role of *rasl11b* maternal expression and suggest that the increase of *rasl11b* expression observed in Z*oep* mutants (Fig. 3H,I) is unlikely a cause of Z*oep* endoderm and PP defects. Besides, *rasl11b* inhibition did not rescue any defects of MZ*oep* mutant, showing that the *oep* maternal expression is required for *rasl11b* knock down to be effective. It is most likely that the total absence of Nodal signaling pathway in MZ*oep* mutant prevents any formation of dorsal mesendoderm and consequently impedes *rasl11b* knock down compensatory action. Finally, these results show that *rasl11b* is a negative modifier that can modulate mesendoderm formation in Z*oep* mutant.

### Rasl11b activation inhibits endodermal and prechordal plate development in Oep deficient embryos

The ability of *rasl11b* knock down to ameliorate Z*oep* phenotypes suggested that *rasl11b* acts as an inhibitor of mesendoderm formation. To test this hypothesis we created two constitutively active Rasl11b molecules by introducing activating mutations based on the tumor-derived RAS mutations G12V and Q61L [58] (Materials and Methods, and Fig. 1A), thereafter both named Rasl11b* because of their similar influence. Rasl11b* mRNAs were injected in MZ*oep*, Z*oep*, wild-type and in *oep^+/−^* embryos (which are phenotypically wild-type). Injection of Rasl11b* had minor effects on wild-type embryos, only occasionally reducing the tail tip (data not shown). It had no effects on MZ*oep* embryos (data not shown), which do not form any endodermal precursors nor PP. However, Rasl11b* strongly exacerbated head/forebrain formation defects in over 80% of the injected Z*oep* (n = 65) and *oep^+/−^* (n = 88) embryos (Figs. 5I–L). While analysis of early markers showed Rasl11b* had no effect in wild-type gastrulae (data not shown), Rasl11b* inhibited the remaining expression of PP and endodermal cell markers in Z*oep* gastrulae (Figs. 5O,T), and reduced the PP size and endodermal cell number in *oep* ^+/−^ gastrulae (Figs. 5P,Q,U,V). Hence, it appears that Rasl11b activity inhibits endoderm and PP formation, but only when *oep* expression is decreased. This data suggests that Rasl11b is an inhibitor of mesendoderm formation antagonized or counteracted by Oep.

### Rasl11b knock down does not rescue other Nodal pathway mutants

The *oep* phenotype is generally believed to be only a consequence of a Nodal signaling decrease, so a simple hypothesis was that Rasl11b acts as an inhibitor of the Nodal pathway. If this were the case, one might predict that *rasl11b* knock down would rescue mesendodermal defects in other Nodal pathway mutants. To investigate this, the effect of *rasl11b* knock down was examined in *cyc*, MZ*sqt*, *bon* and *cas* mutants, which are characterized by a reduction in Nodal signaling and display severe or total absence of PP or endoderm [6], [42], [49], [51]. However, in contrast to Z*oep* mutant, *rasl11b* MO-ATG injection had no effect on any of these mutants. We examined cyclopia, cardia bifida, endodermal cell number and prechordal plate by morphological studies and *in situ* hybridization, yet no rescue was observed (Fig. 6A and data not shown). Similarly, Rasl11b* mRNA injections did not exacerbate defects in *cyc* and MZ*sqt* mutants (data not shown). These results suggest that Rasl11b does not act as an inhibitor of Nodal pathway and that the rescue of Z*oep* mutants by *rasl11b* knock down is not due to a simple compensation for a decrease in Nodal signaling but highlight the specificity of *rasl11b* knock down suppressor effect on the Z*oep* phenotype .

### Rasl11b does not act on Smad2 phosphorylation level

It was still possible that Rasl11b modulates Nodal signaling by acting specifically via Oep. To investigate this, we monitored phosphorylation of the Nodal intracellular signal transducer Smad2 in *oep* mutants with a normal or down regulated expression of *rasl11b*. To detect subtle variations of Smad2 phosphorylation (Smad2-P) level, we wanted to monitor it in each Z*oep* or *rasl11b* MO-ATG; Z*oep* embryo. However, a zebrafish embryo at blastula or gastrula stage does not contain enough material for both a Smad2 phosphorylation assay and DNA extraction to determine its Z*oep* (*oep* ^−/−^) or *oep* ^−/+^ or *oep^+/+^* genotype.

To bypass this limitation we performed our experiments on groups of 100% Z*oep*-like embryos. To generate these 100% homogenous Z*oep*-like groups, we produced clutches of 100% MZ*oep* embryos (by crossing *oep* ^−/−^ fish, see [22] for generation of viable and fertile *oep* ^−/−^ adults) in which we artificially reintroduced an *oep* maternal component by injecting at one cell stage a small quantity of wild-type *oep* mRNA (0.1pg) mimicking perfectly the Z*oep* phenotype (see Materials and Methods). We then compared the number of endodermal cells and Smad2-P level in Z*oep*-like embryos and Z*oep*-like embryos injected with *rasl11b* MO-ATG. Whereas *rasl11b* knock down improved the Z*oep*-like embryo phenotype (similarly to what was observed for Z*oep* embryos), as assayed by *sox17* positive cell number, this had no effect on Smad2 phosphorylation level (Fig. 6B, compare 2^nd^ and 3^rd^ lanes). To account for possible transient or stage-specific effects on Smad2-P, we repeated the Smad2-P detection at different pre-gastrulation stages (Fig. 6C and data not shown) but we never observed any variation of Smad2-P level. It was still possible that the rescue was due to a subtle effect on Smad2, too weak to be detected. To address this possibility, we injected MZ*oep* eggs with a slightly higher dose of wild-type *oep* mRNA (0.2 pg) compared to the dose used to generate Z*oep*-like embryos (0.1 pg). Whereas the number of *sox17* positive cell was smaller in these embryos than in *rasl11b* MO-ATG; Z*oep*-like embryos (mean of 40 vs 47 cells, Fig. 6B), we observed a clear increase of the corresponding Smad2-P level (Fig. 6B, compare 3^rd^ and 4^th^ lanes). Moreover, consistently with this observation, the inhibition of *rasl11b* in MZ*oep* embryos injected with this higher dose of *oep* mRNA further improved their endodermal phenotype but without affecting the Smad2-P level (Fig. 6B, see last lane).

Finally, confirming this data, we never observed during all our experiments any transcript level increase of the intermediate Nodal pathway members downstream of Smad2, i.e. *mixer*, *gata5* and *mezzo,* in Z*oep* or Z*oep*-like embryos injected with *rasl11b* MO-ATG. Altogether, these results demonstrate that *rasl11b* does not act upstream of Smad2 and confirm that Rasl11b does not influence the activation level of the Nodal pathway but rather acts on mesendoderm formation in a Nodal-independent way.

## Discussion

In conclusion, we report here the identification of Rasl11b, a new small GTPase belonging to a poorly studied subgroup of atypical cytoplasmic Ras-related GTPases. *In vivo* functional analyses of Rasl11b revealed the first developmental role for a member of this Ras subgroup. *rasl11b* loss-of-function acts as a specific suppressor of *oep* phenotype showing that Rasl11b can function as a negative modulator of endoderm and prechordal plate formation, and suggesting a Nodal-independent role of Oep on mesendoderm formation.

### Rasl11b belongs to a poorly understood group of cytosolic Ras small GTPase

The Ras family of small GTPases is subdivided into Rho, Rab, Arf and Ras subfamilies. In humans, the Ras subfamily is composed of 35 members classified into 12 structural or functional subgroups (for review see [40]). Rasl11b, together with Rasl11a, Ris/Rasl12 and Rerg, form one of these subgroups. Most Ras subfamily proteins are membrane-localized, due to sequence-specific lipid modifications. These lipid modifications are in turn thought to be responsible for distinct membrane localization characteristics [59], [60] and signaling properties [61] of Ras GTPases. Interestingly, the Rasl11b subgroup is devoid of all standard lipid membrane localization signals. Consistent with this, we have shown that zebrafish Rasl11b localization, like Rerg, is cytosolic. It is possible that this difference in subcellular localization reflects a new type of function for the Rasl11b class of small GTPases. This report is the first to describe a role for a member of this cytosolic Ras GTPase subgroup, Rasl11b, in development. Other studies have implicated a role for Rasl11b subgroup members in oncogenesis. For instance, *rasl11a* mRNA is less abundant in some prostate cancers [38], *ris*/*rasl12* tumor suppressor function in breast cancer is controversial [37], [62], and *rerg* expression inhibits tumor formation in nude mice and is decreased or lost in primary human breast tumors [39]. Thus, clarifying the biological role of this subgroup of small GTPase necessitates further studies.

### Rasl11b is an antagonist of Oep function

We show here that inhibition of *rasl11b* efficiently rescues formation of endoderm, PP and their derivatives in Z*oep* embryos. This rescue is rather dramatic and demonstrates a strong genetic interaction between *rasl11b* and *oep* but it is also remarkable because mutants or loss-of-functions with suppressor abilities have not often been described in zebrafish [63], [64]. One notable feature of *rasl11b* is that, on its own, *rasl11b* loss-of-function appears to have little effect on zebrafish normal development. We found that knock down of *rasl11b* in wild-type embryos caused no major defects save a curly down phenotype. Similarly, *rasl11b* knock down does not have a general ability to rescue mesendodermal defects, as *cyc*, *sqt*, *bon* and *cas* mutant phenotypes were not affected by *rasl11b* knock down. In fact, the effects of Rasl11b knock down and activation appear to be highly specific to animals which have a reduced level or function of Oep. *rasl11b* loss-of-function only suppresses Z*oep* and Rasl11b* enhances Z*oep* phenotype and aggravates *oep* ^+/−^ embryo phenotype (normally wild-type). It is therefore likely that *oep* (but not Nodal) acts to prevent the mesendoderm-inhibiting function of *rasl11b*, whether directly or in parallel. In this model, *rasl11b* role would already be inhibited in a wild-type context, which is consistent with our finding that *rasl11b* knock down has weak phenotype in wild-type embryos. In conclusion, perhaps Rasl11b modifies the output of Oep influence, but only in a context where *oep* expression is decreased. Alternatively, Rasl11b could act in a parallel pathway whose activity is only revealed when *oep* expression is compromised.

### Rasl11b does not act on the currently known Nodal pathway

It is well established that Oep/Frl1/Cripto1 is crucial for Nodal ligand–receptor association and the cascade leading to mesendoderm formation in vertebrates. Thus, our hypothesis was that Rasl11b acts as an inhibitor of the TGFβ/Nodal pathway and hence we explored very carefully the different possibilities. First, if Rasl11b acted upstream or at the level of the Oep/Cyc/Sqt/TGFβ receptor complex, *rasl11b* knock down would have led to an upregulation of Nodal signaling and so to an increase of the Smad2-P level and *mixer*, *mezzo* and *gata5* mRNAs expression. Second, if Rasl11b acted downstream of the Oep/Cyc/Sqt/TGFβ receptor complex, we would have expected to observe a rescue not only of Z*oep* but also at least of the *cyc* and/or *sqt* phenotypes. As no variation of Smad2-P and its downstream targets was observed, and no other Nodal pathway mutant phenotype (*cyc*, *sqt*, *mixer*, *gata5*, *cas*, *foxa2*) is suppressed by *rasl11b* loss-of-function, it is likely that Rasl11b does not act on the Nodal pathway in its currently known organization and composition.

### Rasl11b suggests the existence of a Nodal-independent Oep influence on mesendoderm development

So far, *oep*/*cripto1* has been regarded solely as a component of the Nodal pathway during development. However, Oep/Frl1/Cripto1 and Nodals may have independent functional roles: Nodal inhibits Bone Morphogenetic Protein signaling independently of Cripto1, whereas Oep can affect cell motility independently of Nodals [10], [65]. Here we describe two sets of evidence suggesting that Oep can have a Nodal-independent function in mesendoderm development. First, the large *rasl11b* expression domain in Z*oep* gastrulae, potentially a consequence of the expansion of dorsal mesodermal region previously described in this mutant [56], [57], is not due to a weakening of the Nodal pathway since a reduction in the Nodal ligands (Cyc and Sqt) or an interference with Lefty does not lead to such an expansion. Second, Rasl11b knock down and activation dramatically affect *oep^−/−^* and *oep^+/−^* phenotypes but have no effect at all on the Nodal pathway, strongly suggesting for Oep a role of its own in endoderm and PP development. This data yields the first molecular cue for a Nodal-independent role for Oep on mesendoderm development. Such an *oep*-*rasl11b* interaction could be used, for example, to modulate the mesendodermal specification at a very precise cellular level depending on the local concentration of Oep.

In addition to its role in the Nodal pathway, Oep could influence mesendoderm formation through other signaling pathways. For example, in Xenopus, its orthologue Frl1 has been implicated in the Wnt pathway [29]. Moreover, its mammalian orthologue Cripto1 has also been implicated in oncogenic signaling pathways. Cripto1 is well known to produce hyperplasias and adenocarcinomas in the mammary gland [66]. *cripto1* is also dysregulated in a majority of human carcinomas [66]. In cell culture, Cripto1 binds Glypican 1 and activates the ras/raf/ERK/MAPK and PI3-K/AKT/GSK-3b intracellular signaling pathways [30], [31], [32], both of which are involved in regulating cell proliferation and survival. The precise mechanism by which Cripto1 activity drives oncogenesis remains unclear. However, Rasl11b, a member of a putative tumor suppressor subgroup and only potent when Oep function is compromised, may represent an undescribed link between Cripto1 and the oncogenic process. This study prompts an investigation of a putative role for Rasl11b in oncogenic processes.

Finally, how Oep and Rasl11b interact at the molecular level remain to be established. Oep is an extracellular factor and Rasl11b is cytosolic, making a direct interaction in mesendoderm formation unlikely, except during Oep synthesis and trafficking toward the cytoplasmic membrane. Further studies will be required to identify the intermediate actors and signaling pathways that relate the role of Oep and Rasl11b in the embryo. Here we provide strong evidence that this interaction does not involve Nodal, the main inductive pathway involved in mesendoderm formation, suggesting that other pathways, yet to be identified, are at stake.

## Materials and Methods

### Zebrafish strains

The following mutant alleles were used: *oep^tz57^*, *sqt^cz35^*, *cyc^m294^*, *bon^m425^*, *fau^s26^*, *cas^ta56^*, *mol^tv53a^*. MZ*oep* fertile adult fish were generated as previously described [22]. Crossed together, these fish laid clutches containing 100% of MZ*oep* embryos. Clutches containing 25% of Z*oep* (*oep* ^−/−^) embryos were obtained by crossing *oep^tz57^* heterozygous. Z*oep*-like embryos were generated by microinjection of wild-type *oep* mRNA (0.1pg or 0.2pg) into MZ*oep* eggs.

### Isolation of rasl11b, plasmid construction and directed mutagenesis

*rasl11b* cDNA in pSport was isolated from the same substracted library (embryos with over-activated Nodal signaling versus controls) that previously allowed us to clone the *casanova* gene [42], [67]. The *rasl11b* sequence is deposited at GenBank under the accession number DQ983377. The coding sequence of *rasl11b* was subcloned in the pCS2+ vector for mRNA synthesis. The Morpholino resistant form *rasl11b^MOr^* and the two constitutively activated forms *rasl11b^S32V^*, *rasl11b^Q82L^* were generated as described in the exsite mutagenese kit (Stratagen, La Jolla, CA).

### Sequence analyses

Rasl11b protein analysis was performed with Prosite database http://www.expasy.org and Conserved Domain Database http://www.ncbi.nlm.nih.gov .

### Sequence Accession numbers, alignments and phylogenic tree

Peptide alignments were performed using the ClustalX interface. The phylogenic tree was constructed using the neighbor-joining method. The following peptide sequences were used: Dr Rasl11b, ABI97979; Gg Rasl11b, ENSGALP00000022564; Hs RASL11B, NP_076429; Mm RASL11B, AAH83068; Rn Rasl11b, AAH83755; Tr Rasl11b, NEWSINFRUP00000130569; Xl Rasl11b, DAA02255; Dr Rasl11a, ABK96901; Dr Arf1, AAS92646; Dr Arf2, AAH66632; Dr Cdc42, AAQ97755; Dr K-ras, ABF46832; Dr N-ras, AAB40625; Dr Rab5a, AAH49057; Dr Rab5b, AAH66634; Dr Rab5c, NP_958909; Dr Rab22a, NP_991282; Dr RalA, AAH53216; Dr RalB, AAH78184; Dr Ran, AAB97093; Dr Rap1a, AAH71360; Dr Rap1b, AAQ97994; Dr Rap2b, AAH54999; Dr Rasl12, ENSDARP00000022937; Dr Rerg, XP_701315; Dr Rhoa, NP_997914; Dr Rhog, NP_956334; Dr Rhoh, AAX20135. Dr, *Danio rerio*; Gg, *Gallus gallus*; Hs, *Homo sapiens*; Mm, *Mus musculus*; Rn, *Rattus norvegicus*; Tr, *Takifugu rubripes*; Xl, *Xenopus laevis*.

### Whole-mount in situ hybridization and immunostaining

In situ hybridizations and immunohistochemistry were performed as previously described [68]. For sectioning, embryos were embedded in resin (JB4, Polysciences). For immunohistochemistry, polyclonal antibody against Myc-tag (Anti-Myc Tag, Upstate Biotechnology) was used at 1:500.

### RT-PCR

Total RNA was obtained from staged embryos using TRIzol Reagent (Invitrogen). 1 µg of total RNA was then used to produce cDNA with the StrataScript® First Strand cDNA Synthesis Kit (Stratagene, La Jolla , CA). PCR was performed using the following primers:

1. rasl11b-Forward 5′-ATGcgtctgatccagaacatg-3′;
2. rasl11b-Reverse 5′-gtcacactgaagtgacggtgc-3′;
3. oep-Forward 5′-gtgaaagttggggtttctgg-3′;
4. oep-reverse 5′-ggacattcgactagcgagaact-3′;
5. shh-forward 5′-gactgggtctattacgagtccaaa-3′;
6. shh-reverse 5′-gcctgagttcacgcgagaataaat-3′.

### Morpholino, mRNA and lefty protein microinjection

pCS2+ plasmids containing *rasl11b*, *rasl11b^Q82L^*, *rasl11b^S32V^*, *rasl11b^MOr^*, *myc-rasl11b*, oep, Tar* were linearized with *Not*I and sense RNA transcribed with SP6 RNA polymerase using the mMESSAGE mMACHINE kit (Ambion, Austin, TX). The sequences of the morpholino oligos (Gene Tools, Philomath, OR) used in this paper are: rasl11b-MO-ATG 5′-TTGACATGTTCTGGATCAGACGCAT-3′ and rasl11b-MO-GT 5′-AAACTTACCAACGTTTCTCTCGTAG-3′. *oep*-MO was previously described [69], [70]. 5 silent mismatches were introduced in the MO-ATG targeted sequence to create *rasl11b^MOr^*: 5′-ATGCG(T>A)CT(G>A)AT(C>A)CA(G>A)AA(C>T)ATGTCAA-3′. Lefty injections were performed by injecting Lefty protein solutions at 12.5; 25; 50; 75; 100 and 200 ng/μl in PBS+BSA 0.1% (Recombinant-mLefty1, R&D systems, Minneapolis, MN) between blastomeres at hight, sphere or dome stages.

### Smad2 phosphorylation analysis

Analyses were performed following the standard method with a chemiluminescent detection kit (Amersham Biosciences). Embryos devoid of vitellus were resuspended in a Laemmli/RIPA solution (RIPA: 20 mM Tris pH 8 buffer containing 50 mM NaCl, 50 mM EDTA, 100 mM NaF, 1 mM orthovanadate (NaVO4), 25 mM b-glycerophosphate, 1% NP-40 and a protease inhibitor cocktail (Roche, Nutley, NJ)). Total proteins were separated by 10% polyacrylamide gels and transferred to Hybond ECL membranes (Amesham Biosciences) by electroblotting. Polyclonal antibody against Smad2P (anti-phospho-smad2 antibody, Chemicon International) was used at 1∶500.

### Morpholino efficiency and specificity assay

The *rasl11b* MO-ATG and *rasl11b* MO-GT efficiencies were assayed by in vitro translation and RT-PCR analyses respectively. For in vitro translation, capped *rasl11b* and *rasl11b^MOr^* RNA (20 ng/µl) were translated in vitro in the presence of increasing concentration of MO-ATG (0.04 to 40 µM) using [^35^S] methionine in Rabbit reticulocyte lysate (Promega). RT-PCR was performed as described above on control embryos and MO-GT injected embryos. PCR products were analyzed by electrophoresis and purified for sequencing.

## References

[1] Conlon FL, Lyons KM, Takaesu N, Barth KS, Kispert A (1994). A primary requirement for nodal in the formation and maintenance of the primitive streak in the mouse.. Development.

[2] Zhou X, Sasaki H, Lowe L, Hogan BL, Kuehn MR (1993). Nodal is a novel TGF-beta-like gene expressed in the mouse node during gastrulation.. Nature.

[3] Osada SI, Wright CV (1999). Xenopus nodal-related signaling is essential for mesendodermal patterning during early embryogenesis.. Development.

[4] Rebagliati MR, Toyama R, Fricke C, Haffter P, Dawid IB (1998). Zebrafish nodal-related genes are implicated in axial patterning and establishing left-right asymmetry.. Dev Biol.

[5] Sampath K, Rubinstein AL, Cheng AM, Liang JO, Fekany K (1998). Induction of the zebrafish ventral brain and floorplate requires cyclops/nodal signalling.. Nature.

[6] Feldman B, Gates MA, Egan ES, Dougan ST, Rennebeck G (1998). Zebrafish organizer development and germ-layer formation require nodal-related signals.. Nature.

[7] Alexander J, Stainier DY (1999). A molecular pathway leading to endoderm formation in zebrafish.. Curr Biol.

[8] Willis SA, Zimmerman CM, Li LI, Mathews LS (1996). Formation and activation by phosphorylation of activin receptor complexes.. Mol Endocrinol.

[9] Gray PC, Greenwald J, Blount AL, Kunitake KS, Donaldson CJ (2000). Identification of a binding site on the type II activin receptor for activin and inhibin.. J Biol Chem.

[10] Yeo C, Whitman M (2001). Nodal signals to Smads through Cripto-dependent and Cripto-independent mechanisms.. Mol Cell.

[11] Renucci A, Lemarchandel V, Rosa F (1996). An activated form of type I serine/threonine kinase receptor TARAM-A reveals a specific signalling pathway involved in fish head organiser formation.. Development.

[12] Aoki TO, Mathieu J, Saint-Etienne L, Rebagliati MR, Peyrieras N (2002). Regulation of nodal signalling and mesendoderm formation by TARAM-A, a TGFbeta-related type I receptor.. Dev Biol.

[13] Thisse C, Thisse B (1999). Antivin, a novel and divergent member of the TGFbeta superfamily, negatively regulates mesoderm induction.. Development.

[14] Meno C, Gritsman K, Ohishi S, Ohfuji Y, Heckscher E (1999). Mouse Lefty2 and zebrafish antivin are feedback inhibitors of nodal signaling during vertebrate gastrulation.. Mol Cell.

[15] Meno C, Shimono A, Saijoh Y, Yashiro K, Mochida K (1998). lefty-1 is required for left-right determination as a regulator of lefty-2 and nodal.. Cell.

[16] Cheng AM, Thisse B, Thisse C, Wright CV (2000). The lefty-related factor Xatv acts as a feedback inhibitor of nodal signaling in mesoderm induction and L-R axis development in xenopus.. Development.

[17] Schier AF, Talbot WS (2005). Molecular genetics of axis formation in zebrafish.. Annu Rev Genet.

[18] Tian T, Meng AM (2006). Nodal signals pattern vertebrate embryos.. Cell Mol Life Sci.

[19] Ding J, Yang L, Yan YT, Chen A, Desai N (1998). Cripto is required for correct orientation of the anterior-posterior axis in the mouse embryo.. Nature.

[20] Shen MM, Wang H, Leder P (1997). A differential display strategy identifies Cryptic, a novel EGF-related gene expressed in the axial and lateral mesoderm during mouse gastrulation.. Development.

[21] Kinoshita N, Minshull J, Kirschner MW (1995). The identification of two novel ligands of the FGF receptor by a yeast screening method and their activity in Xenopus development.. Cell.

[22] Gritsman K, Zhang J, Cheng S, Heckscher E, Talbot WS (1999). The EGF-CFC protein one-eyed pinhead is essential for nodal signaling.. Cell.

[23] Zhang J, Talbot WS, Schier AF (1998). Positional cloning identifies zebrafish one-eyed pinhead as a permissive EGF-related ligand required during gastrulation.. Cell.

[24] Xu C, Liguori G, Persico MG, Adamson ED (1999). Abrogation of the Cripto gene in mouse leads to failure of postgastrulation morphogenesis and lack of differentiation of cardiomyocytes.. Development.

[25] Rosa FM (2002). Cripto, a multifunctional partner in signaling: molecular forms and activities.. Sci STKE.

[26] Yan YT, Liu JJ, Luo Y, E C, Haltiwanger RS (2002). Dual roles of Cripto as a ligand and coreceptor in the nodal signaling pathway.. Mol Cell Biol.

[27] Reissmann E, Jornvall H, Blokzijl A, Andersson O, Chang C (2001). The orphan receptor ALK7 and the Activin receptor ALK4 mediate signaling by Nodal proteins during vertebrate development.. Genes Dev.

[28] Kiecker C, Muller F, Wu W, Glinka A, Strahle U (2000). Phenotypic effects in Xenopus and zebrafish suggest that one-eyed pinhead functions as antagonist of BMP signalling.. Mech Dev.

[29] Tao Q, Yokota C, Puck H, Kofron M, Birsoy B (2005). Maternal wnt11 activates the canonical wnt signaling pathway required for axis formation in Xenopus embryos.. Cell.

[30] De Santis ML, Kannan S, Smith GH, Seno M, Bianco C (1997). Cripto-1 inhibits beta-casein expression in mammary epithelial cells through a p21ras-and phosphatidylinositol 3′-kinase-dependent pathway.. Cell Growth Differ.

[31] Bianco C, Strizzi L, Rehman A, Normanno N, Wechselberger C (2003). A Nodal- and ALK4-independent signaling pathway activated by Cripto-1 through Glypican-1 and c-Src.. Cancer Res.

[32] Bianco C, Adkins HB, Wechselberger C, Seno M, Normanno N (2002). Cripto-1 activates nodal- and ALK4-dependent and -independent signaling pathways in mammary epithelial Cells.. Mol Cell Biol.

[33] Kannan S, De Santis M, Lohmeyer M, Riese DJ, Smith GH (1997). Cripto enhances the tyrosine phosphorylation of Shc and activates mitogen-activated protein kinase (MAPK) in mammary epithelial cells.. J Biol Chem.

[34] Brandt R, Normanno N, Gullick WJ, Lin JH, Harkins R (1994). Identification and biological characterization of an epidermal growth factor-related protein: cripto-1.. J Biol Chem.

[35] Minchiotti G, Parisi S, Liguori G, Signore M, Lania G (2000). Membrane-anchorage of Cripto protein by glycosylphosphatidylinositol and its distribution during early mouse development.. Mech Dev.

[36] Normanno N, De Luca A, Bianco C, Maiello MR, Carriero MV (2004). Cripto-1 overexpression leads to enhanced invasiveness and resistance to anoikis in human MCF-7 breast cancer cells.. J Cell Physiol.

[37] Silva J, Silva JM, Barradas M, Garcia JM, Dominguez G (2006). Analysis of the candidate tumor suppressor Ris-1 in primary human breast carcinomas.. Mutat Res.

[38] Louro R, Nakaya HI, Paquola AC, Martins EA, da Silva AM (2004). RASL11A, member of a novel small monomeric GTPase gene family, is down-regulated in prostate tumors.. Biochem Biophys Res Commun.

[39] Finlin BS, Gau CL, Murphy GA, Shao H, Kimel T (2001). RERG is a novel ras-related, estrogen-regulated and growth-inhibitory gene in breast cancer.. J Biol Chem.

[40] Colicelli J (2004). Human RAS superfamily proteins and related GTPases.. Sci STKE.

[41] Barradas M, Gonos ES, Zebedee Z, Kolettas E, Petropoulou C (2002). Identification of a candidate tumor-suppressor gene specifically activated during Ras-induced senescence.. Exp Cell Res.

[42] Dickmeis T, Aanstad P, Clark M, Fischer N, Herwig R (2001). Identification of nodal signaling targets by array analysis of induced complex probes.. Dev Dyn.

[43] Peyrieras N, Strahle U, Rosa F (1998). Conversion of zebrafish blastomeres to an endodermal fate by TGF-beta-related signaling.. Curr Biol.

[44] David NB, Rosa FM (2001). Cell autonomous commitment to an endodermal fate and behaviour by activation of Nodal signalling.. Development.

[45] Aoki TO, David NB, Minchiotti G, Saint-Etienne L, Dickmeis T (2002). Molecular integration of casanova in the Nodal signalling pathway controlling endoderm formation.. Development.

[46] Key MD, Andres DA, Der CJ, Repasky GA (2005). Characterization of RERG: An Estrogen-Regulated Tumor Suppressor Gene.. Methods Enzymol.

[47] Warga RM, Nusslein-Volhard C (1999). Origin and development of the zebrafish endoderm.. Development.

[48] Melby AE, Warga RM, Kimmel CB (1996). Specification of cell fates at the dorsal margin of the zebrafish gastrula.. Development.

[49] Rebagliati MR, Toyama R, Haffter P, Dawid IB (1998). cyclops encodes a nodal-related factor involved in midline signaling.. Proc Natl Acad Sci U S A.

[50] Gritsman K, Talbot WS, Schier AF (2000). Nodal signaling patterns the organizer.. Development.

[51] Kikuchi Y, Trinh LA, Reiter JF, Alexander J, Yelon D (2000). The zebrafish bonnie and clyde gene encodes a Mix family homeodomain protein that regulates the generation of endodermal precursors.. Genes Dev.

[52] Reiter JF, Alexander J, Rodaway A, Yelon D, Patient R (1999). Gata5 is required for the development of the heart and endoderm in zebrafish.. Genes Dev.

[53] Poulain M, Lepage T (2002). Mezzo, a paired-like homeobox protein is an immediate target of Nodal signalling and regulates endoderm specification in zebrafish.. Development.

[54] Norton WH, Mangoli M, Lele Z, Pogoda HM, Diamond B (2005). Monorail/Foxa2 regulates floorplate differentiation and specification of oligodendrocytes, serotonergic raphe neurones and cranial motoneurones.. Development.

[55] Draper BW, Morcos PA, Kimmel CB (2001). Inhibition of zebrafish fgf8 pre-mRNA splicing with morpholino oligos: a quantifiable method for gene knockdown.. Genesis.

[56] Strahle U, Jesuthasan S, Blader P, Garcia-Villalba P, Hatta K (1997). one-eyed pinhead is required for development of the ventral midline of the zebrafish (Danio rerio) neural tube.. Genes Funct.

[57] Schier AF, Neuhauss SC, Helde KA, Talbot WS, Driever W (1997). The one-eyed pinhead gene functions in mesoderm and endoderm formation in zebrafish and interacts with no tail.. Development.

[58] Sekiya T, Fushimi M, Hori H, Hirohashi S, Nishimura S (1984). Molecular cloning and the total nucleotide sequence of the human c-Ha-ras-1 gene activated in a melanoma from a Japanese patient.. Proc Natl Acad Sci U S A.

[59] Prior IA, Harding A, Yan J, Sluimer J, Parton RG (2001). GTP-dependent segregation of H-ras from lipid rafts is required for biological activity.. Nat Cell Biol.

[60] Prior IA, Muncke C, Parton RG, Hancock JF (2003). Direct visualization of Ras proteins in spatially distinct cell surface microdomains.. J Cell Biol.

[61] Hancock JF (2003). Ras proteins: different signals from different locations.. Nat Rev Mol Cell Biol.

[62] Nieto M, Barradas M, Criado LM, Flores JM, Serrano M (2007). Normal cellular senescence and cancer susceptibility in mice genetically deficient in Ras-induced senescence-1 (Ris1).. Oncogene.

[63] Halpern ME, Hatta K, Amacher SL, Talbot WS, Yan YL (1997). Genetic interactions in zebrafish midline development.. Dev Biol.

[64] Etard C, Behra M, Ertzer R, Fischer N, Jesuthasan S (2005). Mutation in the delta-subunit of the nAChR suppresses the muscle defects caused by lack of Dystrophin.. Dev Dyn.

[65] Warga RM, Kane DA (2003). One-eyed pinhead regulates cell motility independent of Squint/Cyclops signaling.. Dev Biol.

[66] Strizzi L, Bianco C, Normanno N, Salomon D (2005). Cripto-1: a multifunctional modulator during embryogenesis and oncogenesis.. Oncogene.

[67] Dickmeis T, Mourrain P, Saint-Etienne L, Fischer N, Aanstad P (2001). A crucial component of the endoderm formation pathway, CASANOVA, is encoded by a novel sox-related gene.. Genes Dev.

[68] Hauptmann G, Gerster T (1994). Two-color whole-mount in situ hybridization to vertebrate and Drosophila embryos.. Trends Genet.

[69] Maroon H, Walshe J, Mahmood R, Kiefer P, Dickson C (2002). Fgf3 and Fgf8 are required together for formation of the otic placode and vesicle.. Development.

[70] Nasevicius A, Ekker SC (2000). Effective targeted gene ‘knockdown’ in zebrafish.. Nat Genet.

